# Injection Molding Plastic Solar Cells

**DOI:** 10.1002/advs.202304720

**Published:** 2023-09-29

**Authors:** Ignasi Burgués‐Ceballos, Paula Pinyol‐Castillo, Aina López‐Porta, Enric Pascual, Tomáš Syrový, Lucie Syrova, Frantisek Josefik, Benjamin Dhuiège, Irene Serrano, Paul D. Lacharmoise, Laura López‐Mir

**Affiliations:** ^1^ EURECAT Technology Centre of Catalonia Functional Printing and Embedded Devices Unit Parc Científic TecnoCampus Av. Ernest Lluch 36 Mataró 08302 Spain; ^2^ EURECAT Technology Centre of Catalonia Polymeric and Composites Processes Unit Parc Tecnològic del Vallès Av. Universitat Autònoma, 23 Cerdanyola del Vallès 08290 Spain; ^3^ Faculty of Chemical Technology Department of Graphic Arts and Photophysics University of Pardubice Doubravice 41 Pardubice 53353 Czech Republic; ^4^ Centre for Organic Chemistry 296 Rybitví Rybitví 533 54 Czech Republic; ^5^ GenesInk 39 Avenue Gaston Imbert Zi De Rousset Rousset 13790 France; ^6^ Aitiip Engineering Department Polígono Industrial Empresarium Calle Romero 12 Zaragoza 50720 Spain

**Keywords:** in‐molds, organic photovoltaics, plastic solar cells, solution‐processing, upscaling electronics

## Abstract

While organic photovoltaics are accessing specific application sectors taking advantage of their unique properties, it is important to identify as many differentiators as possible to expand the market penetration and consolidation of this technology. In this work, for the first time, the large‐scale fabrication of organic photovoltaic modules embedded into structural plastic parts through industrial injection molding is demonstrated. Thermoplastic polyurethane is chosen as the injected material to show that this additional processing step can yield flexible, lightweight photovoltaic modules with enhanced device robustness and virtually unchanged performance. The critical optomechanical and physico‐chemical material properties, as well as the plastic processing parameters to enable in‐mold plastic solar cells with improved performance and stability, are discussed and provided with perspective.

## Graphical abstract

Organic photovoltaic modules embedded into plastic parts through high throughput injection molding are demonstrated here for the first time. The modules injected with thermoplastic polyurethane show enhanced mechanical stability while keeping a high flexibility, with neglectable efficiency losses and a remarkable process yield close to 90%.

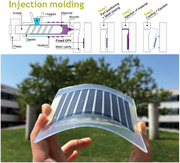

## 1 Introduction

To accelerate the market penetration of organic photovoltaics (OPV), it is important that both R&D and the commercialization roadmap focus on specific application areas that exploit the distinct advantages of OPV.^[^ ^1^ ^]^ Paradigmatic examples include transparent OPV for power‐generating windows^[^ ^2^, ^3^ ^]^ and building‐integrated photovoltaics,^[^ ^4^ ^]^ wavelength‐selective absorption for agrivoltaics,^[^ ^5^, ^6^ ^]^ high indoor and low‐light efficiency for low‐power and IoT applications,^[^ ^7^, ^8^ ^]^ and flexibility and washability for wearable electronics.^[^ ^9^, ^10^ ^]^ While the vast majority of those developments pursue the maximization of the optoelectronic properties of OPV, little attention has been paid to their structural properties. High‐volume manufacturing technologies such as plastic injection molding can help expand the opportunities, the capabilities, and the seamless integration of OPV.

Due to their very thin layout, flexible solar cells can be sensitive to mechanical abrasiveness and, therefore, might require additional protection and integration strategies. Such conventional strategies typically include adhesion to rigid surfaces or attachment to additional bulky structures with frames and contacts. In that respect, embedding a printed solar module into a plastic part simplifies these integration challenges, while providing additional mechanical protection, shape adaptability, and streamlined contacts for connections. The concept behind in‐mold photovoltaics is highly innovative and rather unexplored, with very few works so far reporting on over‐molding amorphous silicon‐based^[^ ^11^ ^]^ and CIGS (Copper Indium Gallium Selenide)‐based photovoltaics.^[^ ^12^ ^]^ These two photovoltaic materials suffered a significant loss in power conversion efficiency upon injection molding. Yet, the technological maturity of in‐mold electronics, currently demonstrated in relatively simple integrated circuits and components,^[^ ^13^ ^]^ encourages further research to realize more complex in‐mold optoelectronic devices such as organic solar cells.

Here, we present the first flexible organic solar cell modules embedded into 3D plastic parts through injection molding. The aim of this work is to demonstrate the high potential of in‐mold organic photovoltaics (IM‐OPV) and their compatibility with large‐scale production. The whole fabrication process, including roll‐to‐roll wet‐processing and injection molding, was carried out in industrial processing lines under ambient conditions. We conducted thorough analyses of the optoelectronic and mechanical performance of the modules before and after injection, as well as of their operational stability. We adapted the injection molding process to obtain a yield of ∼90%, which lays the first stone of IM‐OPV as a promising technology for niche applications.

## 2 Results and Discussion

### 2.1 Large‐scale Fabrication of Fully Solution‐Processed, Organic Photovoltaic Modules

In this study, we fabricated a short series of fully printed solar cell modules in an industrial roll‐to‐roll line following previously described procedures.^[^ ^14^, ^15^, ^16^ ^]^ The flexible OPV modules were processed in an ambient atmosphere, prioritizing the use of low‐cost materials and low‐energy processing methods. Importantly, the choice of materials for the fabrication of the modules also considered two critical aspects for product development: i) proven compatibility with high throughput, roll‐to‐roll processing, thus including rather thick layers with high tolerance to variations, and ii) the specific requirements to withstand the injection molding process, which entails a short exposure to high temperature, pressure, and shear stress (Figure S1, Supporting Information). Hence, we purposely avoided the use of brittle materials such as indium tin oxide (ITO), and the highest‐performing donor–acceptor blends that have not yet demonstrated enough intrinsic and morphological stability under thermal and light stress.^[^ ^17^ ^]^ Instead, highly flexible and stable materials are much preferred for the IM‐OPV application herein proposed.

**Figure 1:**
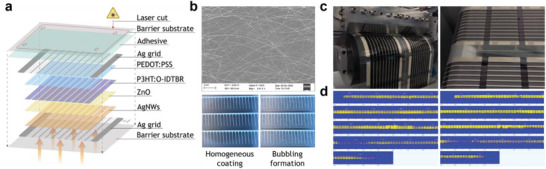
In‐line fabrication and characterization of organic photovoltaic modules. a) Outline of the complete single‐junction stack. b) Appearance of the Ag nanowires layer: (top) SEM top view image, (bottom) two close‐up photographs of Ag NWs layers on top of the front Ag grid, comparing a homogeneous coverage against bubbling formation. c) Pictures of the roll‐to‐roll fabrication of the photovoltaic modules. The processing techniques used include slot‐die coating, rotary screen printing, and flexography. d) Panoramic LBIC images of 330 modules measured with a contactless, in‐line characterization system.^[^ ^22^ ^].^

For that reason, we opted for a module stack incorporating the workhorse P3HT:O‐IDTBR photoactive blend, as it has been successfully used in roll‐to‐roll production lines and has demonstrated morphological and thermal stability,^[^ ^18^, ^19^ ^]^ as well as semitransparent electrodes based on silver grids. A schematic of the whole stack is shown in **Figure** **1a**. First, we designed a front electrode inspired by the *flextrode* concept^[^ ^20^, ^21^ ^]^ consisting of a silver grid covered with a semitransparent layer of custom‐made silver nanowires developed by GenesInk. The Ag combs had a length and a pitch distance of 10 mm and 2 mm, respectively. The Ag nanowires ink dispersion was specifically designed for rotary screen printing, and the printing conditions were adjusted to yield high‐quality coverage of the grids. For instance, simply by reducing the printing speed from 4 to 3 mm s^−1^, we successfully prevented the formation of bubbles in the Ag nanowires layer (Figure 1b), presumably due to a reduced shear stress. Second, we sequentially slot‐die coated the ZnO (electron transport), P3HT:O‐IDTBR (active), and PEDOT:PSS (hole transport) layers on top of the front electrode (Figure 1c). The custom‐made PEDOT:PSS ink dispersion was specifically designed to work both as the hole transport layer and as the back electrode, in combination with the flexography printed back Ag grid. Each module had 10 interconnected sub‐cells and a total active area of 40 cm^2^. All the modules were subjected to an in‐line electrical characterization prior to the roll‐to‐roll UV‐lamination process.

Any roll‐to‐roll printing process requires a certain degree of losses while running (i.e., web distance) to reach the web speed and printing settings that yield the desired registration and homogeneous coating of the layers. Consequently, it is particularly challenging in a multi‐layer stack such as that of the OPV modules to track the regions of the foil that contain the optimally printed layers. In this respect, the implementation of high throughput imaging techniques that assist such identification is of high value. Herein, we used a roll‐to‐roll light beam‐induced current (LBIC) system to find the web sections hosting the highest‐performing OPV modules. This powerful analytical tool collects medium‐resolution, photocurrent maps of the modules in a contactless manner at a web speed of 1 m min^−1^. Figure 1d shows the panoramic LBIC characterization of a section containing 330 modules, from where 64 modules were selected (i.e., laser cut) to carry out all the studies presented in this study. This set of large‐area, flexible modules exhibited an average power conversion efficiency of 4.45 ± 0.36%, with a *V_OC_* of 7.09 ± 0.06 V, an *I_SC_* of 35.7 ± 2.2 mA, a FF of 61.8 ± 2.6%, and a maximum power output (*P_MPP_*) of 154 ± 10 mW.

### 2.2 Injection Molding

**Figure 2:**
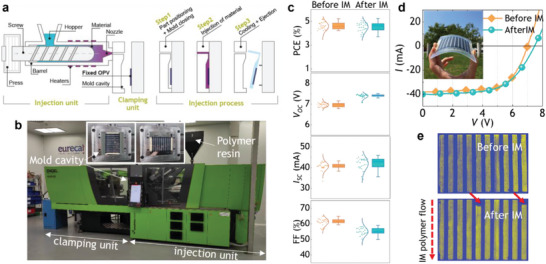
Injection molding processing of OPV modules. a) Schematics of the injection molding process. b) The Engel COMBI Victory 1050H/200 W/200L injection molding machine used in this study. The inset shows close‐up photographs of the mold cavity holding an OPV module in a vertical (left) or horizontal (right) position. c) Statistical performance data taken from 32 injected modules. All data points are included (left); box range 25/75th percentiles (right). d) *I–V* characteristics of an OPV module before and after injection (see inset picture), showing no loss in performance. e) High‐resolution LBIC images of the module shown in (d) before IM (top) and after IM (bottom). The dashed red arrow indicates the direction of the injected molten polymer; the red solid arrows point to areas of the module that show degradation upon IM.

Injection molding is a transformative manufacturing process widely used for producing plastic components. It involves injecting molten plastic material into a mold cavity under high pressure (**Figure** **2a**), allowing complex shapes with good accuracy and repeatability.^[^ ^23^ ^]^ This section presents a set of experiments focused on the influence of key parameters, such as injection speed or holding pressure, on the functionality of the OPV modules. The findings contribute to the understanding of the applicability of OPV in injection molding, offering practical insights on parameter optimization.

A polyether copolymer‐based thermoplastic polyurethane (TPU) was chosen for the injection molding of OPVs due to its low process temperature, broad substrate compatibility, and flexibility. TPU is a copolymer comprising alternating hard and soft segments. The hard segments provide rigidity and strength, while the soft segments, consisting of polyols like polyester or polyether, contribute to the material's flexibility and elasticity. This copolymer structure enables a balance of key mechanical properties, combining toughness, resilience, and good elongation and recovery characteristics. The selected grade, Pearlthane® Clear 15N80 by Lubrizol, was chosen for its optical properties (i.e., high transparency) and hydrolysis resistance. The process temperatures for this specific grade range from 180–190 °C, thus favoring that the samples retain their functionality upon injection molding with minimal losses, as explained later.

Injecting functional, thin film substrates presents certain challenges, including ink wash‐out, substrate wrinkling, and film‐to‐cavity subjection method.^[^ ^24^ ^]^ Each case requires an in‐depth study to determine the optimal parameters that allow a correct injection process while maintaining the (optoelectronic) functionality of the sample. This applies to IM‐OPV as well, with the added requirement of maintaining a high transparency in the plastic part. For a successful IM process, parameters like injection speed, switchover position, holding pressure and barrel temperatures need to be optimized, and might vary depending on the geometry of the part, as well as on the machine and materials used.

The first viability injections were done using a 120 × 120 × 2 mm cavity insert, attaching the substrate to the mold's cavity with double‐sided tape. Initially, the OPV modules were attached in vertical position, so the molten resin flow followed the long side of the rectangular substrate (see inset in Figure 2b). We observed the appearance of wrinkles at the edge of the substrate sample, which we ascribed to vibrations from the front flow. To overcome this effect, we followed two strategies. First, the injection speed was raised from 60 to 90 mm ^−1^s. By filling the part faster, the turbulence generated in the polymer flow had less time to wrinkle the piece. The polymer's shear application time on the substrate was also reduced. However, based on this, one could also argue that increasing the speed increases the shear value, which leads to the second strategy: the OPV modules were positioned horizontally; although this position increased the sample perimeter at the material inlet, the time during which the material exerted shear on it as well as the resulting turbulence were significantly reduced. This sample position also provided more surface area for adhesive application and, therefore, enabled better adhesion. Additionally, the sample may slide downward slightly during injection due to the shear of the TPU. This effect caused wrinkling in the final part of the vertically oriented samples as they were hitting the cavity wall, whereas no apparent damage was observed in the horizontally oriented samples.

Thanks to the low processing temperature of TPU, wash‐out is not as concerning as when injecting other materials with higher melting temperatures such as polycarbonate. Some samples experienced warping due to the different levels of shrinkage between the substrate and the injected material. This was circumvented by increasing the cooling time and allowing the part to shrink and relax inside the cavity. With these adjustments, we successfully prepared a set of IM‐OPV modules with TPU in two separate experiments using identical processing settings. From the selection of 64 roll‐to‐roll printed modules, 32 of them were injected, and the other 32 modules were kept as references for different kinds of mechanical stress and accelerated degradation tests (see next section).

The performance of the OPV modules before and after the IM process was characterized by standard *I–V* testing under AM 1.5 G illumination. The statistical data collected are summarized in Figure 2c and in **Table** **1**. On average, the IM‐OPV modules retained 98.1 ± 6.5% of the original performance. Only 2 samples failed, and 28 samples preserved ≥ 90% of the original performance, which sets the yield of the IM process close to 90%.

**Table 1:** Average photovoltaic performance parameters of 32 OPV modules under AM 1.5G illumination (1 sun) before and after injection molding (IM).

	*V_OC_* [V]	*I_SC_* [mA]	FF [%]	*V_MPP_* [V]	*I_MPP_* [mA]	*P_MPP_* [mW]	PCE [%]
Before IM	6.95 ± 0.09 **7.00**	40.7 ± 1.8 **38.3**	61.1 ± 2.1 **63.9**	5.20 ± 0.10 **5.08**	33.3 ± 2.2 **33.7**	173 ± 12 **171**	4.58 ± 0.35 **4.38**
After IM	7.40 ± 0.10 **7.47**	41.9 ± 2.8 **40.3**	55.0 ± 2.6 **58.3**	5.11 ± 0.19 **5.24**	33.4 ± 2.5 **33.5**	171 ± 15 **176**	4.52 ± 0.39 **4.49**

Note: open‐circuit voltage (*V_OC_*), short‐circuit current (*I_SC_*), fill factor (FF), maximum power point voltage (*V_MPP_*), maximum power point current (*I_MPP_*), maximum power output (*P_MPP_*), and power conversion efficiency (PCE) with respect to the active area (∼40 cm^2^). The values in bold correspond to a representative device, shown in Figure 1d,e.

Although the power conversion efficiency of the modules did not suffer significant changes upon injection molding, clear trends are observed in the statistical *I–V* data. The average open circuit voltage (*V_OC_*) increased by almost 450 mV, while the fill factor showed a moderate but statistically relevant decrease from 61.1% to 55.0%. In contrast, the average short‐circuit current (*I_SC_*) remained almost unchanged, with a broader data distribution observed after IM. These changes can be seen in Figure 2d, where the *I–V* characteristics of a representative OPV module showcase the observed trends. We ascribe the improvement in *V_OC_* to an enhanced interface contact resulting from the high peak pressure and temperature suffered during the IM process (Figure S1, Supporting Information). The UV–Vis spectra (Figure S2, Supporting Information) reveal no changes in the position, width, or relative intensity of the absorption bands. Therefore, we discarded the idea that this particular IM process could induce significant morphological changes in the photoactive layer, such as prolonged crystallization of P3HT or O‐IDTBR, or severe phase segregation.^[^ ^25^ ^]^ The high‐resolution LBIC imaging characterization (Figure 2e and Figure S3, Supporting Information) revealed a somehow hidden side effect of the IM process. Looking at the photocurrent maps, we observed systematically small regions in the active area with no or drastically diminished current generation (indicated with red arrows in Figure 2e). These degraded areas were always located at the top of the sample, coinciding with the first point where the molten resin front hits the module while being held in the mold cavity. As explained above, this is typically a critical point in an IM process when a thin film sample is smaller than the mold cavity: the polymer fills homogeneously the cavity downwards until it reaches the edge of the film; at that point the shear stress and pressure increase as the section is reduced, which could damage locally the OPV modules. This explanation is supported by our performed simulations that show the highest shear stress at the top edge of the OPV module (Figure S1, Supporting Information). Consequently, the effective active area of the IM‐OPV modules was partially lowered, which would intuitively result in lower photocurrent generation. Surprisingly, the hypothesized interface improvement upon IM could be responsible for the observed increase in photocurrent generation across the active module area (see the more homogeneous LBIC images in bottom Figure 2e and Figure S3, Supporting Information). This unexpected, positive side effect compensated for the loss in the active area: the average *I_SC_* was statistically constant. Considering the high cost of building molds for IM with custom dimensions, alternative strategies that allow placing samples with different dimensions in a given mold without compromising their performance would be of high relevance.

### 2.3 Mechanical and Operational Stability

**Figure 3:**
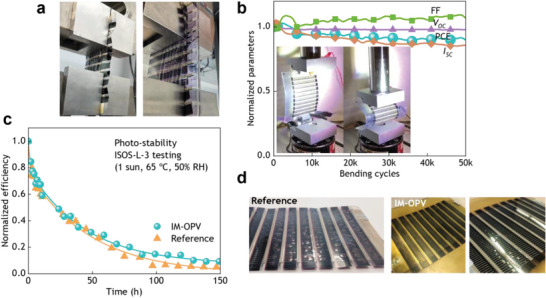
Mechanical stress and accelerated degradation testing. a) Photographs of control (left) and in‐mold (right) OPV module subjected to uniaxial tensile stress until delamination. b) *I–V* derived performance parameters of an IM‐OPV module as a function of bending cycles. c) Efficiency evolution of 2 modules subjected to accelerated photo‐stability degradation according to ISOS‐L‐3. d) Close‐up photographs of the degraded modules in (c), showing a much lower bubbling formation and yellow coloration on the IM‐OPV modules.

To confirm the enhancement in the mechanical properties of the IM‐OPV modules thanks to the injected TPU plastic, generic stress tests were carried out. Uniaxial tensile testing (**Figure** **3a**) revealed an average >35% increase in the maximum stress point in the IM‐OPV samples with respect to the control devices (**Table** **2**). Noteworthy, the first fracture on the control devices occurred at 10–30% of strain, whereas this value jumped up to 70–150% on the IM‐OPV modules, owing to the significantly higher deformability of TPU. Similarly, due to the elastomeric nature of TPU, delamination of the IM‐OPV modules occurred before reaching the ultimate strength point. On the other hand, no physical damage nor efficiency loss was observed when exposing the IM‐OPV modules to flexural stress (Figure S4, Supporting Information) due to the high flexibility of TPU.

**Table 2:** Strain‐stress points of interest obtained with the tensile testing.

	Maximum stress point, *s_m_* [MPa]	Strain at maximum stress point [%]	Strain at fracture, *e_b_* [%]
Control OPV module	670 ± 21	69 ± 29	82 ± 29
IM‐OPV module	919 ± 152	109 ± 27	263 ± 111

When subjected to cyclic bending stress, a power conversion efficiency retention higher than 90% was found after 50 000 cycles with a bending radius of 27 mm (Figure 3b). These data validate the choice of TPU as the injected material, with the IM‐OPV modules showing higher mechanical stability while maintaining a reasonably high flexibility.

In addition to the enhanced mechanical strength obtained with the IM‐OPV modules, we investigated whether the injected TPU provided any benefit in terms of extra sealing that would result in longer operational stability. We subjected a set of modules (both injected and not‐injected) to accelerated degradation testing, following the harshest protocols for photo‐stability (i.e., ISOS‐L‐3: 1 sun, 65 °C, 50% of relative humidity) and thermal stability (i.e, ISOS‐D‐3: dark conditions, 65 °C, 85% of relative humidity).^[^ ^26^, ^27^ ^]^ The data collected from the photo‐stability ISOS‐L‐3 tests revealed no significant differences in the progressive efficiency loss of the injected and not‐injected modules. The resulting exponential decay occurred all along the degradation process, that is, no stabilization was reached in these devices (Figure 3c). The corresponding *T_80_* (the time when performance reaches 80% of the initial performance) was determined at 2.5 h in both cases, which falls within the expected range of lifetimes under the ISOS‐L‐3 degradation conditions.^[^ ^28^ ^]^ Noteworthy, a yellow coloration appeared on the IM‐OPV modules upon constant 1 sun illumination (Figure 3d). This unwanted yellowing is a result of UV irradiation,^[^ ^29^ ^]^ therefore it could be potentially circumvented by using another TPU grade composition that includes UV‐blocking additives. A significantly lower density of bubbles was observed in the degraded IM‐OPV samples (Figure 3d), which points to a diminished delamination resulting from an enhanced adhesion. On the contrary, the degradation of the IM‐OPV modules under the ISOS‐D‐3 (damp heat testing) conditions was slightly faster than the reference devices (Figure S5, Supporting Information). The reason for this small difference in thermal stability is yet to be determined. We hypothesize that TPU could introduce a higher degree of mechanical stress due to differences in thermal expansion coefficients with the PET substrate. Overall, these results encourage further exploration of alternative injection materials or additives to provide the IM‐OPV modules with enhanced thermal and light stability.

## 3 Conclusion

We have developed organic photovoltaic modules embedded into plastic parts through high throughput injection molding. We have successfully adapted the industrial plastic processing conditions to obtain in‐mold modules with neglectable efficiency losses and a remarkable process yield close to 90%. Our results remind us that OPV product development demands photovoltaic materials with high morphological stability under thermal stress, such as the P3HT:O‐IDTBR blend. In that respect, higher‐performing materials with such stability are urgently needed. We also stress the importance of the injected material to expand the mechanical properties of in‐mold OPV modules. The injected thermoplastic polyurethane enhances its mechanical stability while keeping a high flexibility. We believe that future focus on injection plastic materials could further extend the benefits of in‐mold photovoltaics in regards to structural and device stability, or even providing additional optical functionalities. This work represents the first demonstration of in‐mold plastic solar cells and opens new possibilities for organic photovoltaics to enable specific applications that require simultaneous high optoelectronic and structural performances.

## 4 Experimental Section

### Materials

Unless stated otherwise, the solvents and chemicals used in this study were obtained commercially and used without further purification. The modules were prepared on PET‐based barrier material as described in the literature.^[^ ^16^ ^]^ The Ag nanowires and the PEDOT:PSS dispersions were self‐synthesized (see sections below). A nanoparticle‐based ZnO ink in an alcohol solvent (ZnO ink, from infinityPV ApS) was used for the electron transport layer. The photoactive ink consisted of P3HT (from Rieke Metals) and O‐IDTBR (from 1‐Material). The photoactive materials were mixed in a 1:1 ratio and dissolved in chlorobenzene with 5% vol. chloronaphthalene (both solvents from Sigma‐Aldrich) in a total concentration of 40 mg/ml. The solution was stirred at 500 rpm at 80 °C for 1 h prior to its use in the slot‐die coating process. A UV‐curable, epoxy‐based adhesive (X‐5, from infinityPV ApS) was used for the lamination of the printed modules. A thermoplastic polyurethane (Pearlthane Clear 15N80, from Lubrizol) was used for the injection molding process.

### Preparation of Ag Nanowires Formulation

The Ag nanowires ink formulation was developed by GenesInk under the name TranDuctive Screen C47NSCR02041. The ink was composed of 0.2% wt. of silver nanowires in a mixture of solvents, mainly water, alcohol, and glycol, with cellulose derivative additives as rheological agents. A patent submitted in 2022 protects the ink formulation. The ink was specially designed to be suitable for screen printing. Indeed, the ink has a shear‐thinning property with viscosity in the range of 1–2, 0.8–1.8, 0.8–1.4 Pa.s at shear rates of 10, 40, and 100 s^−1^, respectively. Once deposited by Dr Blade with 24 µm wet thickness onto flexible substrate, e.g., polyethylene terephthalate (PET), the transparent conductive layer based on silver nanowires showed i) a sheet resistance of 40 ± 15 Ω/≤ measured with a 4‐probe instrument, ii) a transmittance at 550 nm superior to 90% measured with UV–Vis spectroscopy, and iii) an adhesion of 5B using the ASTM D3359 standard method. The stability of the ink had been validated over 9 months at 4 °C and at room temperature.

### Preparation of PEDOT:PSS Solution

The water‐based dispersion of PEDOT:PSS was prepared in a 1:2.5 ratio as follows. To a solution of sodium polystyrene sulfonate (*M* = 1 000 000 g mol^−1^, 12.9 g, 62.56 mmol) in demineralized water (500 ml), FeCl_3_.6H_2_O (50 mg, 0.185 mmol), (NH_4_)_2_S_2_O_8_ (6.84 g, 29.98 mmol) and 3,4‐ethylenedioxythiophene (3.56 g, 25.04 mmol) were added. The reaction mixture was stirred and heated at 45 °C for 24 h. The reaction mixture was purified by ion exchange with an acidic and a basic ion exchanger. Then, subsequent purification steps such as ultrafiltration were performed. The product concentration was adjusted with demineralized water to a dry solid content of 1%. The ink formulation for slot‐die coating was based on freshly synthesized PEDOT:PSS, consisting of 188.2 g of the PEDOT:PSS 1:2.5 dispersion, to which 0.211 g of polyoxyethylene octyl phenyl ether‐based surfactant were added dropwise under mixing with magnetic stirrer. After 1 h of mixing, 9.890 g of ethylene glycol was added drop by drop using a peristaltic pump under mixing at 800 rpm. Under the same conditions, 7.40 g of isopropyl alcohol was added. The whole ink was further mixed for 2 h. The ink formulation was then filtrated through 0.2 µm PTFE filters. With the resulting ink formulation, transparent conductive films were prepared and characterized, providing a specific conductivity of 724 S cm^−1^, determined with a four‐point probe and mechanical profilometry (Tencor P‐7).

### Roll‐to‐Roll Fabrication of Organic Photovoltaic Modules

The complete stack of the fully solution‐processed modules was PET‐based barrier/Ag grid/Ag nanowires/ZnO/P3HT:O‐IDTBR/PEDOT:PSS/Ag grid/X‐5 adhesive/PET‐based barrier (Figure 1A). The devices were fabricated using roll‐to‐roll processing as described in the literature.^[^ ^16^ ^]^ The custom‐made Ag nanowire ink was rotary screen printed at a rather low speed of 3 m min^−1^ to prevent bubbling formation (see Figure 1A). The ZnO, P3HT:O‐IDTBR, and custom‐made PEDOT:PSS inks were sequentially slot die coated using a 20 stripe mask (defining 2 modules within the web width) at a web speed of 10, 1.5, and 20 m min^−1^, and wet thicknesses of 6.25, 10.4, and 37 µm, respectively. The silverback electrode was flexo printed at a web speed of 2 m min^−1^. After roll‐to‐roll electrical characterization, the completed modules were laminated, and characterized post‐lamination with a high throughput roll‐to‐roll LBIC system, (Antikythera from infinityPV ApS), and subsequently laser cut into individual parts.

### Injection molding of Organic Photovoltaic Modules

Thermoplastic polyurethane was injected according to the standard processing recommendations from the resin supplier using an Engel COMBI VC 1050H/200 W/200L​ machinery through a 120 × 120 × 2.5 mm mold cavity (Figure 2B). The clamping force was fixed at 600 kN, and the injection molding parameters were optimized to lower in intensity and shorten in time the peak temperature and peak pressure as well as the shear stress on the OPV modules. Specifically, the injection temperature, injection speed, and holding pressure time were tuned in the ranges of 185 °C to 200 °C, 60 to 90 mm ^−1^s, and 5 to 10 s, respectively. To obtain high‐quality injected plastic parts it was also essential to fine‐tune the switchover from first to second stage injection. This critical parameter was not standardized across machines, and it was highly dependent on the geometry of each mold cavity (and on the OPV module dimensions, in this case). Overall, the total injection time and peak pressure were lower than 2 s and 90 bar, respectively. The total process time for each IM‐OPV module, including the two injection stages, holding times, and cooling time was ≈60 s.

### Measurements

The performance of the modules before and after injection was characterized with accurate *I–V*‐testing and calibrated light sources (AM 1.5 G under ambient conditions) in two independent setups (ISOSun, infinityPV ApS, and Atlas SolarTest 1200, with a CalCell reference device, infinityPV ApS). The *I–V* characteristics were recorded using a Keithley 2602A SourceMeter and an automatic multiplexer. Non‐destructive light beam‐induced current (LBIC) mapping was performed in two steps: first, rapid screening of all the modules in the foil was performed with high throughput, contactless, roll‐to‐roll system (Antikythera R2R LBIC from infinityPV ApS);^[^ ^22^, ^30^ ^]^ second, high‐resolution LBIC images were taken on a desktop system (Professional LBIC from infinityPV ApS). 4 injected modules and 2 reference modules were used for each of the ISOS tests as well as for the mechanical stress evaluation. For ISOS‐D‐3 the samples were placed in an environmental chamber (from CCi) set at 85% relative humidity (RH) and 65°C chamber temperature. The modules were kept at open circuit conditions and periodically tested under a halogen lamp illumination. For ISOS‐L‐3 a xenon lamp‐based weathering chamber (Suntest XXL+ from Atlas) was set to 65 °C air temperature, 85 °C device temperature (black panel), 50% RH, and illumination of 1 Sun, and the modules were kept at maximum power point load between measurements. The *I–V*‐curve tracing of the modules was performed using an automated acquisition setup with a multiplexer and a Keithley 2602A SourceMeter. The recorded *I–V* curves were used to construct the degradation curves as recommended in the ISOS protocols.^[^ ^26^ ^]^ The bending tests were conducted in an MTS Landmark Servoghydr test system with customized clamps to hold the modules, which were cycled up to 50 000 times between a relaxed position and a vertical displacement of 70 mm or 100 mm, corresponding to bending radii of 27 and 20 mm, respectively. The *I–V* characteristics were recorded every 100 bending cycles using a solar simulator (from Ossila) and a Keithley 2602A SourceMeter. The tensile tests were performed under ambient conditions with a universal test machine (Zwick Roell Z 2.5 10 kN ProLine MPMS S0206, from Zwick GmbH) following the ISO 527‐2/5A/200 test method.

### Statistical Analysis

All the raw data points obtained from the 32 IM‐OPV modules were included in the statistical analysis without pre‐processing. Those data points are shown in Figure 2c, together with boxes that indicate the mean (center line), median (center empty square), and 25/75th percentile (bottom/top lines) values, with the outliers containing the rest of the data points. The analysis and presentation of the statistical data were performed with Origin (OriginLab).

## Conflict of Interest

The authors declare no conflict of interest.

## Author Contributions

I.B.‐C. and L.L.‐M. conceived the idea and designed the experiments. I.B.‐C., P.P.‐C., and L.L.‐M fabricated all the solar cell samples, conducted the measurements, and performed data analysis. A.L.‐P. and E.P. led the injection molding experiments. T.S., L.S., and F.J. developed and supplied the PEDOT:PSS formulations. B.D. developed and supplied the Ag nanowires dispersion. I.S. conducted mechanical stress analyses. P.D.L. contributed to the discussion of the results. L.L.‐M. directed the project. I.B.‐C., A.L.‐P., and L.L.‐M. wrote the manuscript with input from all co‐authors.

## Supporting information

Supporting Information

## Data Availability

The data that support the findings of this study are available from the corresponding author upon reasonable request.
