# Supplementary material for: Injection Molding Plastic Solar Cells
Source: Adv Sci (Weinh). 2023 Sep 29;10(32):2304720. doi: 10.1002/advs.202304720 (PMC10646225; doi:10.1002/advs.202304720)
Supplement: Supplementary file 1 — Supporting Information [file ADVS-10-2304720-s001.pdf]

ADVANCED SCIENCE
Open Access

Supporting Information

for *Adv. Sci.*, DOI 10.1002/advs.202304720

Injection Molding Plastic Solar Cells

Ignasi Burgués-Ceballos, Paula Pinyol-Castillo, Aina López-Porta, Enric Pascual, Tomáš Syrový, Lucie Syrova, Frantisek Josefik, Benjamin Dhuiège, Irene Serrano, Paul D. Lacharmoise and Laura López-Mir*

Supporting Information

Injection molding plastic solar cells

Ignasi Burgués-Ceballos, Paula Pinyol-Castillo, Aina López-Porta, Enric Pascual, Tomáš Syrový, Lucie Syrova, Frantisek Josefik, Benjamin Dhuiège, Irene Serrano, Paul D. Lacharmoise, Laura López-Mir*

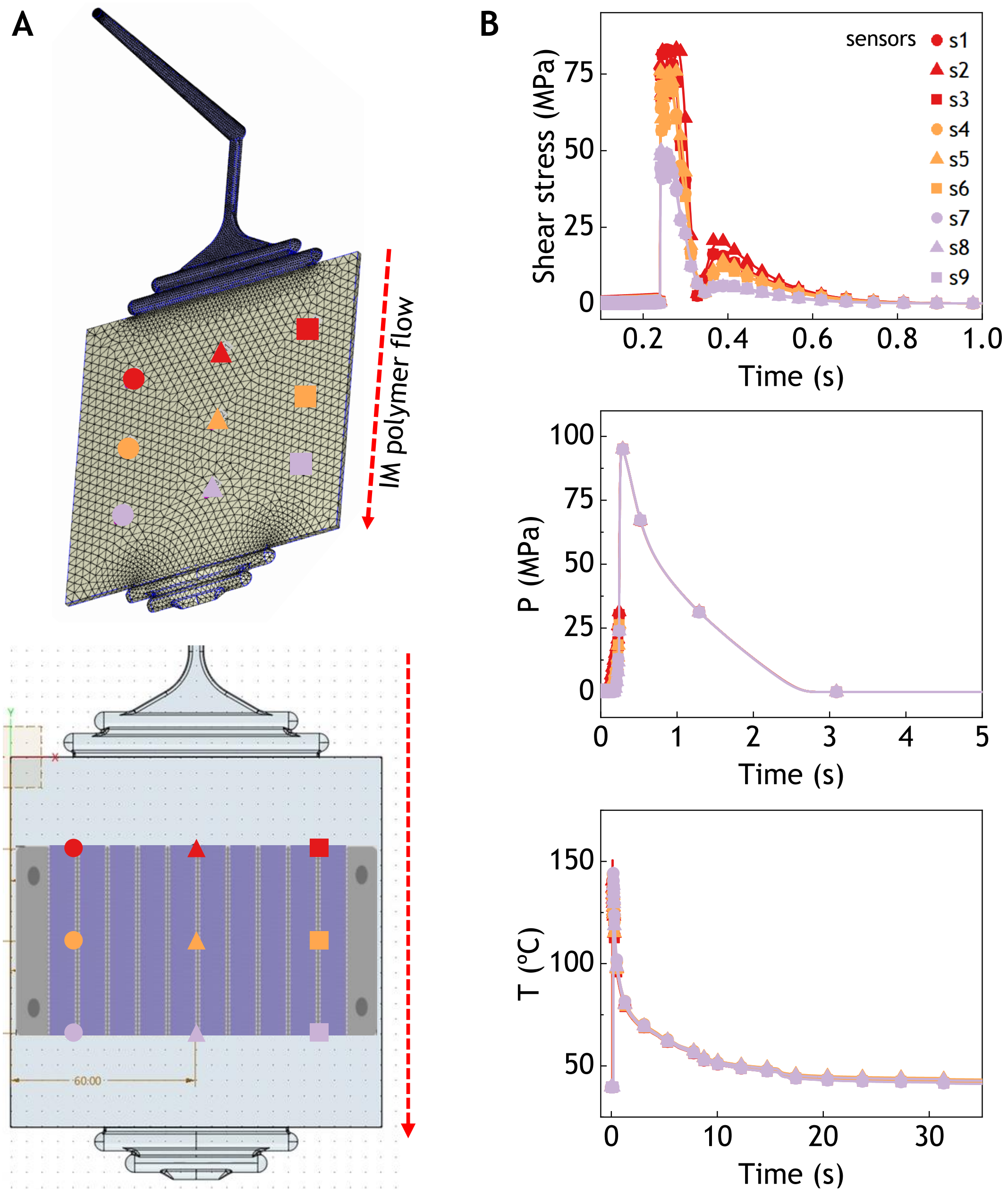

Figure S1. Simulation of stress during injection molding. a) Sketches of the injected plastic part with 9 sensors (circles, triangles and squares), and the OPV module in horizontal position

(bottom sketch). b) Simulated shear stress, pressure and temperature over time upon injection molding. The top 3 sensors suffer the highest peak shear stress.

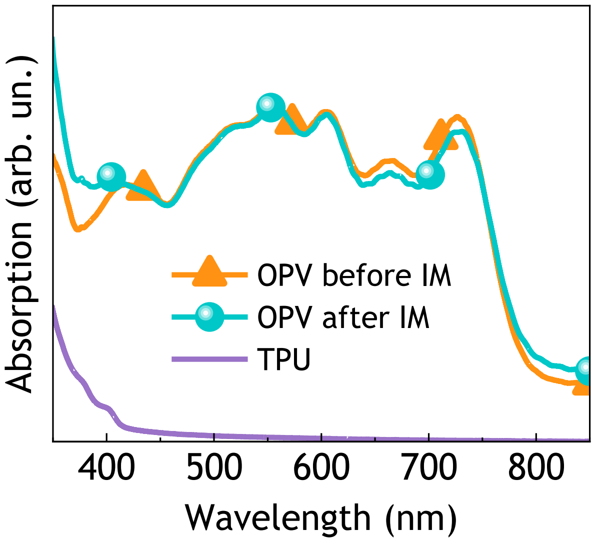

Figure S2. UV-Vis spectra of an organic photovoltaic module before and after the injection molding process, together with the absorption of the injected TPU polymer. The two module spectra show no changes in peak position, width or relative intensity, thus suggesting no morphological changes.

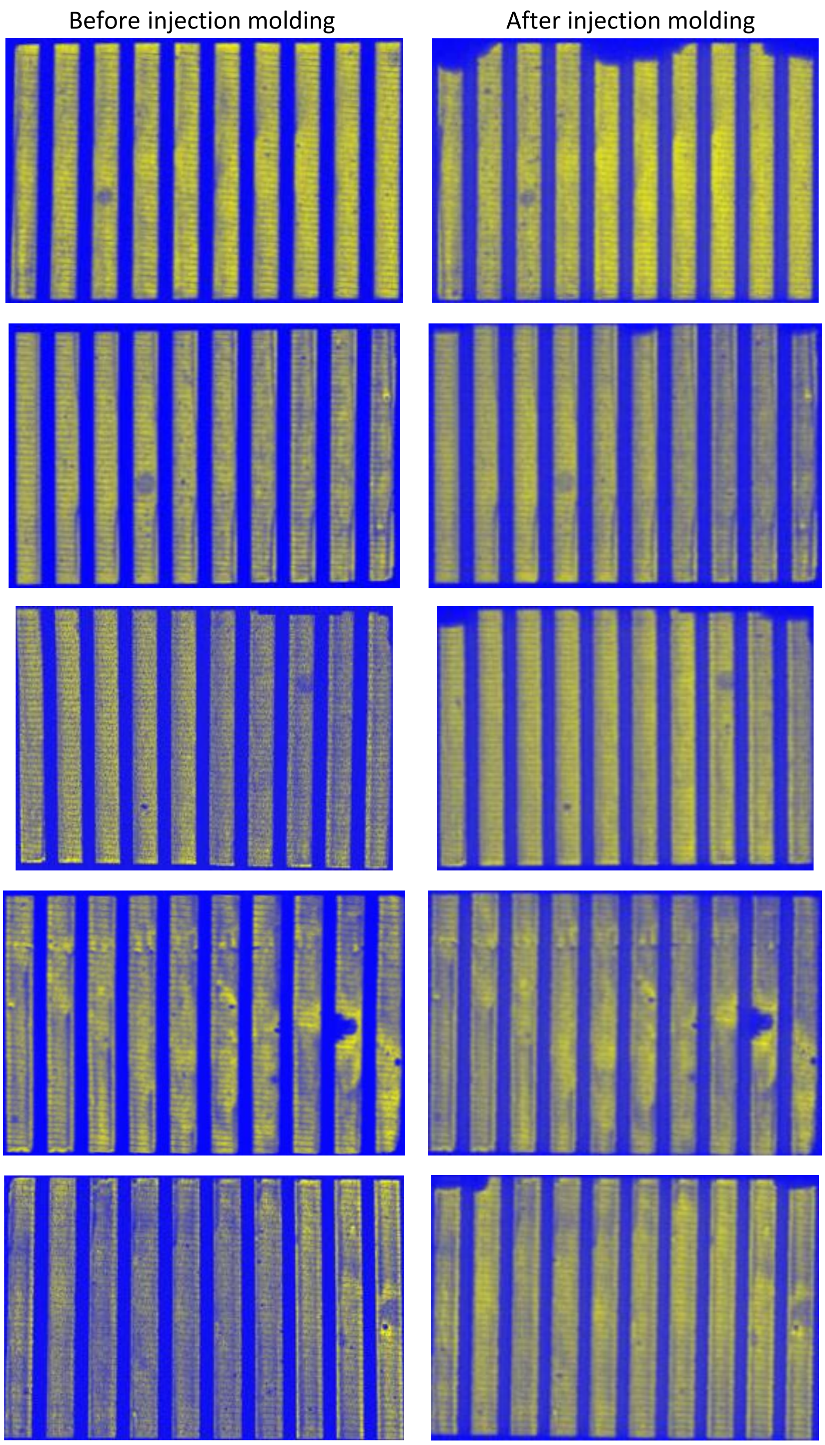

Figure S3. High resolution LBIC images of several modules before (left) and after (right) the inmold process. Some of these IM-OPV modules presented, as the one shown in Figure 2E, damaged areas at the top edge due to the higher shear stress from the molten polymer. On the contrary, a more intense and homogeneous photocurrent generation was observed in the IM-OPV modules.

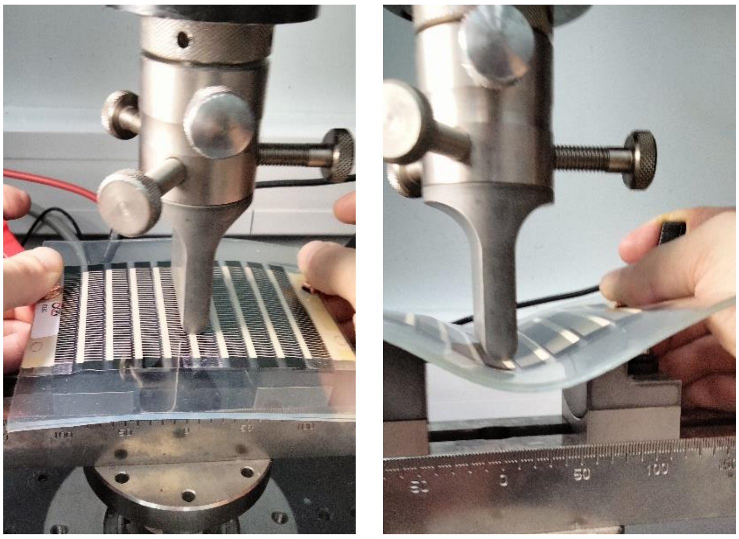

Figure S4. The flexural stress testing did not cause mechanical nor functional losses to the IM-OPV modules.

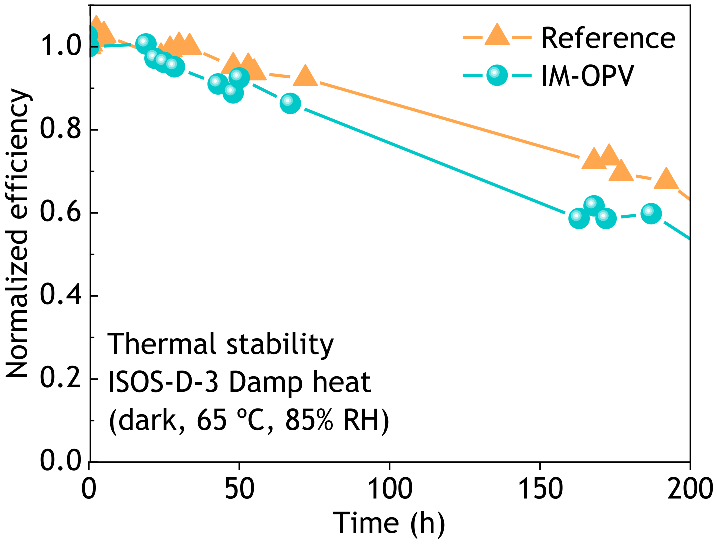

Figure S5. Accelerated thermal degradation of a reference and an IM-OPV module under the ISOS-D-3 testing protocol conditions.
